# A novel role for microglia in minimizing excitotoxicity

**DOI:** 10.1186/1741-7007-10-7

**Published:** 2012-01-31

**Authors:** Mariko L Howe, Ben A Barres

**Affiliations:** 1 Department of Neurobiology, Stanford University School of Medicine, Stanford, CA 94305, USA

## Abstract

Microglia are the abundant, resident myeloid cells of the central nervous system (CNS) that become rapidly activated in response to injury or inflammation. While most studies of microglia focus on this phenomenon, little is known about the function of 'resting' microglia, which possess fine, branching cellular processes. Biber and colleagues, in a recent paper in *Journal of Neuroinflammation*, report that ramified microglia can limit excitotoxicity, an important insight for understanding mechanisms that limit neuron death in CNS disease.

See research articlehttp://www.jneuroinflammation.com/content/9/1/27.

## Commentary

Constituting as many as 10% of cells in the central nervous system (CNS), microglia are a subset of glial cells whose function is a longstanding mystery. Unlike other glial cells - the astrocytes and oligodendrocytes - microglia derive from cells of the immune system. As such, they act as first responders to direct CNS damage and peripheral insults, a response characterized by rapid hypertrophy of their long fine cell processes, upregulation of cytokine and surface receptor expression, and increased phagocytosis [1]. The morphological changes associated with their transformation from ramified 'resting' microglia to ameboid 'activated' microglia were reported in the 19th century based on observations of CNS tissue infected with rabies virus, from a deceased multiple sclerosis patient, and later in neurodegenerative diseases [2]. Since then, many studies have focused on the importance of activated microglia in health and disease. The function of ramified microglia, however, remains elusive. Two photon *in vivo *imaging reveals that fluorescently labeled ramified microglia constantly extend and retract processes, preferentially contacting synapses in an activity-dependent manner [3]. Following these studies, many have called for rebranding 'resting' microglia as 'monitoring' microglia or some more descriptive appellation in keeping with our increasing understanding of what they do. A recent study by Knut Biber and colleagues in the *Journal of Neuroinflammation *now significantly furthers our grasp of microglial function by demonstrating a neuroprotective role of microglia in N-methyl-D-aspartic acid (NMDA)-induced excitotoxicity [4].

**Figure 1:**
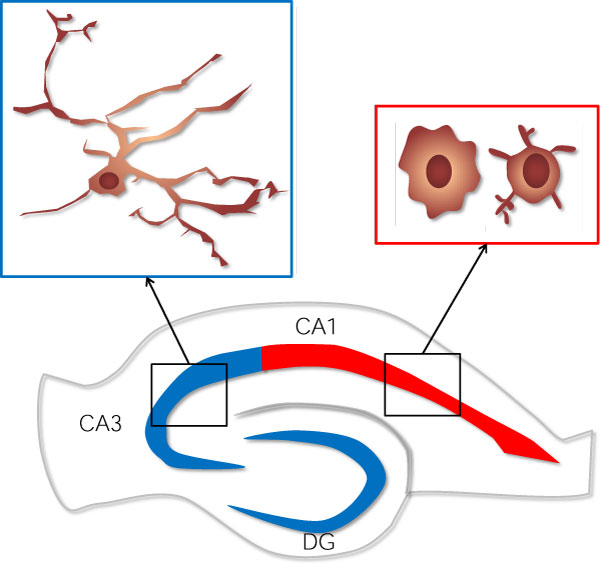
**Differential susceptibility of hippocampal subregions to NMDA-induced excitotoxicity is correlated with microglial morphology**. CA1 neurons (red) are most vulnerable to excitotoxicity, compared with CA3 and dentate gyrus (DG) neurons (blue). In response to NMDA challenge, microglia within CA1 adopt an activated morphology, whilst microglia within protected regions retain a ramified appearance (insets).

NMDA receptors are a subset of glutamate receptors, which can lead to neuronal excitation; too much glutamate, such as after CNS damage, can promote excessive excitation and become toxic. Treatment of mouse organotypic hippocampal slice cultures with micromolar amounts of NMDA induces neuronal cell death in a region-specific manner. Neurons of the CA1 region are most susceptible to NMDA-induced toxicity, whereas neurons in CA3 and dentate gyrus (DG) are less vulnerable. The reason for this differential vulnerability is unclear, although Vinet *et al. *[4] noticed that there are more morphologically activated microglia within CA1, an area of high neuronal death, compared with CA3 and DG (Figure 1). To determine the effect of microglia on NMDA-dependent neurotoxicity, they ablated microglia within hippocampal slices using two methods - clodronate liposomes and a genetically encoded thymidine kinase that induces apoptosis of proliferating cells upon gancyclovir treatment - and assessed the level of neuronal cell death. Surprisingly, loss of microglia exacerbated NMDA-induced toxicity, abolishing the differential vulnerability of neurons in hippocampal subregions at some NMDA doses. These data suggested a neuroprotective function of microglia. The researchers tested this possibility by reseeding microglia-depleted slices with primary microglia from mixed glial cultures before NMDA treatment. These microglia successfully engrafted, exhibiting branching, fine processes similar to ramified microglia in the healthy CNS. Remarkably, repletion of microglia resulted in significant neuroprotection compared with slice cultures maintained without microglia.

This study demonstrates a highly neuroprotective function of microglia in NMDA-induced excitotoxicity and suggests that ramified microglia may contribute to this neuroprotection. The findings raise several important questions. First, what is the relationship between microglial morphology and function? While much is known about stimuli that promote microglial activation, relatively little is understood about the function and mechanism of the ramified morphology. The CNS milieu likely contributes, as bone marrow-derived monocytes co-cultured with astrocytes adopt a ramified, microglia-like appearance [5]. In addition, disruption of microglia-neuron interactions, such as those mediated by the cell surface protein CD200 on neurons and its receptor on microglia, results in abnormal microglial morphology [6]. Importantly, donor-derived monocytes infiltrate the CNS following bone marrow transplantation and eventually adopt a ramified, microglia-like morphology [7]. However, can such blood-derived cells truly become microglia? During development microglia derive from primitive macrophages that enter the developing neural tube during early embroygenesis and, except in injury like irradiation for bone marrow transplantation, are not replaced by infiltrating bone marrow-derived monocytes [7]. In addition, increasing evidence suggests divergent functions for these cell types in mouse models of disease. To fully appreciate the mysterious function of microglia in health and disease, a better understanding of the relationship between microglial morphology and function is needed.

Second, what is the precise mechanism by which microglia confer protection from NMDA-induced excitotoxicity? Vinet *et al. *[4] report that ramified microglia contribute to reduced neurotoxicity based on the observations that ramified microglia appear in less vulnerable hippocampal regions and that replenished microglia prevent excitotoxicity and eventually become ramified. It is also possible that these microglia - which by virtue of the culture conditions become activated - release neuroprotective factors. Alternatively, microglia may secrete as yet unidentified NMDA antagonists or secrete cytokines that regulate NMDA receptor function or density. In fact, TNFalpha, a cytokine produced exclusively by microglia and macrophages within the brain, has previously been shown to regulate levels of another class of neuronal glutamate receptors called AMPA receptors [8]. Additionally, Biber's group previously reported that the chemokine CXCL10 produced by astrocytes may mediate NMDA-induced neurotoxicity via CXCR3 signaling in microglia [9]. It is possible that microglia signal to nearby astrocytes to control their levels of glutamate uptake transporters. Whatever the mechanism, the finding that microglia powerfully control excitotoxicity provides an important clue for future studies aimed at understanding the molecular mechanisms that control excitotoxicity and how neurons can be better protected in various acute and chronic neurological diseases.

A final critical question raised by the experiments of Vinet *et al. *is whether this type of microglia-mediated neuroprotection occurs *in vivo*. Resident cells of the intact CNS are not exposed to serum. Gene profiling experiments by our lab demonstrate that serum exposure in culture can induce gene expression changes in astrocytes that mimic those of activated astrocytes [10]. Many components of serum are known modulators of microglial activation [2]. In addition, culture in serum often requires non-physiological drug dosages, making *in vivo *translation difficult. While organotypic hippocampal slice cultures have proven extremely valuable in understanding fundamental mechanisms underlying glutamate excitotoxicity, *in vivo *investigation is required to sort out the physiological relevance of these processes in a developing, mature, or injured CNS. Recent advances in imaging technologies, transplantation experiments and genetic ablation have improved our understanding of the importance of microglia in health and disease. However, development of new tools to specifically manipulate microglia *in vivo *is now essential for continued discovery and innovation in microglial research.
